# Glue-assisted exfoliation of two-dimensional sulfur-rich niobium thiophosphate (Nb_4_P_2_S_21_) for sulfur-equivalent electrode study in lithium storage†

**DOI:** 10.1039/d4na01060d

**Published:** 2025-01-30

**Authors:** Bing Wu, Vlastimil Mazánek, Min Li, Martin Veselý, Qiliang Wei, Luxa Jan, Filipa M. Oliveira, Lei Zheng, Heng Li, Vojtech Kundrat, Jakub Zálešák, Jakub Regner, Rui Gusmão, Junjie He, Tomáš Hartman, Saeed Ashtiani, Yulong Ying, Zdenek Sofer

**Affiliations:** a Department of Inorganic Chemistry, University of Chemistry and Technology Prague Technická 5 166 28 Prague Czech Republic wui@vscht.cz zdenek.sofer@vscht.cz; b Department of Physical and Macromolecular Chemistry, Faculty of Science, Charles University in Prague Prague 12843 Czech Republic; c Department of Organic Technology, University of Chemistry and Technology Prague Technická 5 166 28 Prague Czech Republic; d Institute of Micro/Nano Materials and Devices, Ningbo University of Technology Ningbo 315211 P.R. China; e Department of Molecular Chemistry and Materials Science, Weizmann Institute of Science Rehovot 7610001 Israel; f Chemistry and Physics of Materials, University of Salzburg Jakob-Haringer-Strasse 2A 5020 Salzburg Austria; g School of Materials Science and Engineering, Zhejiang Sci-Tech University Hangzhou 310018 PR China

## Abstract

Two-dimensional (2D) layered thiophosphates have garnered attention for advanced battery technology due to their open ionic diffusion channels, high capacity, and unique catalytic properties. However, their potential in energy storage applications remains largely unexplored. In this study, we report a 2D transition metal thiophosphate (Nb_4_P_2_S_21_) with high sulfur content, synthesized *via* chemical vapor transport (CVT). The bulk material, exhibiting a layered quasi-one-dimensional (quasi-1D) structure, can be exfoliated into high-quality nanoplates using glue-assisted grinding. Density functional theory (DFT) calculations reveal a direct bandgap of 1.64 eV (HSE06 method) for Nb_4_P_2_S_21_, aligning with its near-infrared (NIR) photoluminescence at 755 nm. Despite an initial discharge capacity of 1500 mA h g^−1^, the material shows low reversible capacity and rapid capacity decay at 0–2.6 V. *In situ* Raman confirms the formation of polysulfides during cycling. Given its high sulfur content, the material was evaluated at 0.5–2.6 V, 1.0–2.6 V, and 1.5–2.6 V to assess its sulfur-equivalent cathode performance. In carbonate-based electrolytes, electrochemical performance is hindered by polysulfide formation and side reactions, but switching to ether-based electrolytes improves initial reversible capacity and coulombic efficiency due to additional Li_*x*_S conversion above 2.2 V. EDS and TOF-SIMS analyses of cycled electrodes show a significant sulfur loss, worsening the polysulfide shuttle effect and leading to battery failure. Adapting strategies from lithium–sulfur batteries, such as polar host catalysts, could enhance the material's performance.

## Abstract 2

This study uses glue-assisted exfoliation to obtain 2D Nb_4_P_2_S_21_ nanoplates, showing promising low-voltage cathode performance and enhanced electrochemical activity in ether-based electrolytes, despite some shuttle effect challenges.

## Graphical abstract

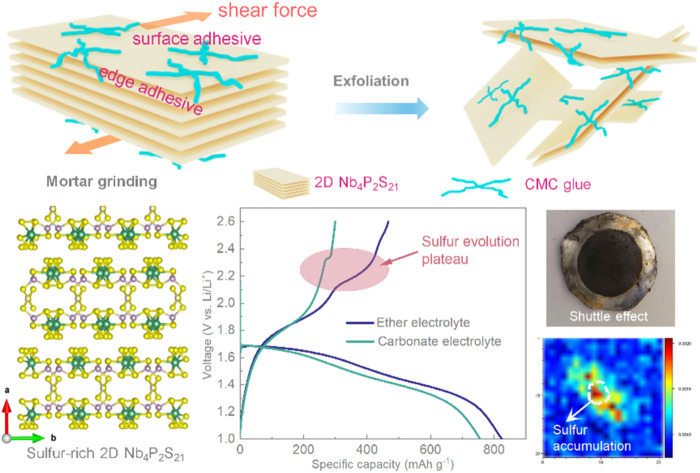

## 1. Introduction

The quest for sustainable and high-performance energy storage systems has become a cornerstone challenge in the field of materials science and engineering.^1^ Lithium-ion batteries (LIBs) have powered a technological revolution, driving portable electronics and electric vehicles. However, the growing demand for energy storage, driven by renewable energy and transport electrification, has exposed limitations in current LIB technology, such as limited energy density, long charging times, and safety risks. These challenges have spurred research into novel materials offering higher capacity, faster ion transport, and improved thermal stability.^2^

Two-dimensional (2D) materials have emerged as leading candidates to address these challenges, with research over the past decade demonstrating their potential to revolutionize energy storage.^3^ These materials, defined by their atomically thin structure and high surface-to-volume ratio, have shown exceptional promise due to their unique physicochemical properties. Moreover, their layered structure facilitates van der Waals interactions that allow for efficient ion intercalation and deintercalation, a crucial mechanism for battery operation. Theoretical and experimental studies have highlighted their ability to facilitate fast electron and ion transport, and offer novel mechanisms for charge storage.^4^ These materials, including graphene, transition metal dichalcogenides, and hexagonal boron nitride, each contribute to a deeper understanding of 2D physics and chemistry while pushing the boundaries of energy storage capabilities.^5^

Within the realm of 2D materials, layered thiophosphates are particularly noteworthy. These materials are part of a broader family of transition metal phosphorus trichalcogenides MPCh_3_ (M = transition metal, Ch = S, Se), which have been extensively studied for their electronic and magnetic properties.^6^ Recent advances highlight the burgeoning potential of these MPCh_3_ materials in the domain of energy storage.^7^ The relationships between the structural characteristics of these thiophosphates and their electrochemical performance underscore the importance of the van der Waals gap and electronic conductivity in dictating the rate capabilities and cycling stability of the resulting batteries. They are recognized for their utility as (1) electrode materials for alkali metal-ion batteries, exemplified by FePS_3_, FePSe_3_, SnP_2_S_6_ and high-entropy (CoVMnFeZn)PS_3_ in lithium/sodium/potassium-ion systems;^7*d*,8^ (2) catalysts facilitating swift conversion of metal (poly)sulfides in metal–sulfur batteries, with bimetallic (FeMn)PS_3_ and (FeCo)PS_3_ being a case in point;^7*c*,9^ and (3) solid-state electrolytes in multivalent metal batteries, as seen with ZnPS_3_ as zinc-ion solid electrolytes.^7*b*,10^ It is worth noting that recently the layered van der Waals material V_2_PS_10_ has gained attention due to its unique properties in lithium and magnesium-ion batteries.^11^ However, other 2D thiophosphate compounds with varying phosphorus and sulfur content, such as Nb_4_P_2_S_21_, may offer distinct advantages for battery applications similar to V_2_PS_10_, yet they remain unreported. The sulfur-rich composition of these materials provides a high theoretical capacity as a result of the multiple electron-exchange reactions that sulfur atoms can undergo. Additionally, materials with rich sulfur structures, such as MoS_*x*_ (3 ≤ *x* ≤ 7),^12^ NbS_*x*_ (3 ≤ *x* ≤ 5),^13^ (Fe/Co/Ni)_2_S_7_,^14^ Co_2_S_9_,^14^ Fe_3_S_8_,^14^ WS_5_,^15^ and the P_4_S_10+*n*_ series,^16^ often exhibit advantages similar to those in lithium–sulfur batteries as sulfur-equivalent electrode materials. These include a high voltage plateau, faster electron conduction in active materials, and more rapid reaction kinetics between alkali metal and active materials. Additionally, such materials, when subjected to high cutoff voltages in LIBs, may not fully discharge, leading to the formation of metallic compounds (such as non-stoichiometric metal sulfides) other than Li_2_S. These compounds can catalyze the subsequent conversion of Li_2_S, functioning similarly to catalysts in lithium–sulfur battery systems. Consequently, they are considered promising high-capacity sulfur-equivalent cathode materials for metal–sulfur battery systems.^17^

Building on this foundation, our research introduces the niobium thiophosphate Nb_4_P_2_S_21_, a sulfur-rich 2D material synthesized through an innovative chemical vapor transport (CVT) process. By employing density functional theory (DFT) calculations, we reveal the direct band gap feature of this material with 1.64 eV (based on the HSE06 method), as well as exhibiting near-infrared (NIR) photoluminescence at 755 nm. The synthesis and subsequent delamination of this material into high-quality nanoplates *via* glue-assisted grinding exfoliation represent a significant breakthrough. This method not only retains the structural integrity of the thiophosphate but also makes the benefits of its narrow 2D layered structure accessible for ion diffusion, leading to potentially superior rate performance over conventional materials. Prior research has underscored the challenge of high sulfur content in electrode materials, particularly concerning electrolyte interactions and the stability of the cathode during cycling. Our study addresses these challenges by presenting a detailed electrochemical analysis of Nb_4_P_2_S_21_, examining its performance across different voltage windows and comparing it with various electrolyte systems from carbonate-based to ether-based electrolytes. Post-cycling failure analysis of the batteries using X-ray diffraction (XRD), Scanning Electron Microscopy/Energy Dispersive X-ray spectroscopy (SEM-EDS), and TOF-SIMS identified the polysulfide shuttle effect, like that in lithium–sulfur batteries, which involves the migration of soluble lithium polysulfides formed during discharge between the anode and cathode, leading to capacity loss. The presented results not only shed light on the electrochemical performance but also guide further enhancements for sulfur-rich materials.

## 2. Experimental section

### 2.1 Growth of Nb_4_P_2_S_21_ single crystal

Nb_4_P_2_S_21_ crystal was synthesized using high-purity elements: niobium powder (99.999%, STREM, Germany), sulfur powder (99.999%, STREM, Germany), and red phosphorus powder (99.999%, STREM, Germany). Phosphorus and sulfur were used in 1 at% excess over stoichiometry. The powders were mixed and sealed in a quartz ampoule under a high vacuum using an oxygen/hydrogen welding torch. The ampoule was positioned in a dual-zone furnace designed for crystal growth. The process was conducted over 7 days for the reaction and formation of Nb_4_P_2_S_21_, maintaining a temperature gradient of 50 °C between the source zone (650 °C) and the growth zone (600 °C). After the heating period, the sample was allowed to naturally cool to room temperature. This synthesis process yielded deep-red needle crystals of Nb_4_P_2_S_21_.

### 2.2 Apparatus

The X-ray diffraction (XRD) pattern was captured using a Bruker D8 X-ray diffractometer, employing Cu Kα radiation (*λ* = 0.15418 nm, *U* = 40 kV, *I* = 40 mA). The surface composition of the samples was further studied with X-ray photoelectron spectroscopy (XPS) using SPECS spectrometer equipped with a monochromatic Al Kα X-ray source (1486.7 eV) and a hemispherical electron analyzer Phoibos 150. Scanning electron microscopy (SEM), scanning transmission electron microscopy (STEM), and energy dispersive spectroscopy (EDS) analyses were conducted using a Tescan MAIA 3 system, integrated with an Oxford EDS detector and operated *via* Aztec software. Thickness measurements of exfoliated nanoplates were performed using Atomic Force Microscopy (AFM) with an Ntegra Spectra instrument from NT-MDT. Raman spectra were obtained using a Renishaw inVia Raman Microscope, featuring backscattering geometry, a CCD camera detector, and a 532 nm, 50 mW DPSS laser at full power. A 20× objective lens was used for sample focusing. Photoluminescence spectra were acquired using a Renishaw InVia Raman confocal microscope, employing a 532 nm excitation, 50 mW Nd-YAG laser. Both bulk and exfoliated suspension were drop-casted onto nickel support for drying. Transmission electron microscopy (TEM) analysis, using a Jeol 2200 FS microscope (Japan) with an attached Oxford Instrument energy dispersive spectroscopy (EDS) system (England), was employed to characterize the crystal structure, morphology, and elemental composition. TF Helios 5 FX dual-beam microscope equipped with a compustage holder and a 256 × 256 px Timepix detector was utilized for a cross-sectional lamella preparation and subsequent electron diffraction analysis. The lamella was prepared in a standard lift-out procedure using ion beam voltages ranging from 30 kV to 2 kV and ion beam currents ranging from 20 nA to 20 pA. The electron diffraction pattern was recorded with the Timepix detector in a spot mode using an electron beam current of 25 pA and electron beam accelerating voltage of 30 kV.

### 2.3 Exfoliation of thin-layered Nb_4_P_2_S_21_ nanoplates

Typically, 0.2 g of bulk crystal and 20 g of 5 wt% carboxymethyl cellulose (CMC, Penta, viscosity 1500–4500 mPa s with 1%[dried substance] in water) glue solution in H_2_O were mixed and ground in a mortar for 45 minutes. The ground mixture was then diluted in 150 mL of deionized water (DI) and centrifuged at 10 000 rpm (10 733 g). The resulting precipitate was repeatedly washed five times with DI to remove any glue residue. Finally, the exfoliated sample was dispersed in dimethylformamide (DMF) solution for further use.

For comparison, the bulk powder was obtained through the same procedure, excluding the addition of CMC glue solution.

### 2.4 Coin-cell battery assembly and measurements

A slurry, homogeneously mixed with exfoliated Nb_4_P_2_S_21_ (active material), carbon black, and polyvinylidene fluoride (PVDF) in a weight ratio of 8 : 1 : 1, was prepared in *N*-methyl pyrrolidone (NMP). This slurry was then evenly applied to a copper foil and subjected to vacuum drying at 80 °C. Subsequently, electrodes of 10 mm diameter were produced, featuring an areal mass loading of 1.0 to 1.2 mg cm^−2^. These electrodes were utilized to construct CR2032 coin cells, paired with lithium foil as the counter electrodes and a Celgard-3501 membrane as the separator. The electrolyte used was 1 M LiPF_6_ in a 1 : 1 volume ratio mixture of ethylene carbonate (EC) and diethyl carbonate (DEC).

Electrochemical testing included discharge/charge performance analysis using a Neware battery test system (Neware BTS 8.0, Shenzhen, China). Additionally, cyclic voltammetry (CV) and electrochemical impedance spectroscopy (EIS) measurements were conducted at room temperature, employing a CorrTest CS Studio electrochemical workstation (Wuhan CorrTest Instrument Corp).

### 2.5 *In situ* Raman measurements

The *in situ* Raman of the battery during charge and discharge was characterized using a two-electrode cell with an optical window. The Raman spectra were collected using a Renishaw inVia Raman Microscope, featuring backscattering geometry, a CCD camera detector, and a 785 nm. An AutoLab PGSTAT204 (Eco Chemie, Utrecht, Netherlands) with NOVA Version 2.1.7 software was employed to perform the discharge/charge process of two electrode cell systems. Fig. S1† presents the *in situ* Raman cell setup used to analyze changes in the Nb_4_P_2_S_21_ electrode.

### 2.6 Electronic structure calculation

The ground state calculations were conducted using the Vienna *Ab initio* Simulation Package (VASP).^18^ The Perdew–Burke–Ernzerhof (PBE) functional^19^ was employed for the exchange–correlation interaction, except for specific band structure calculations, where the HSE06 functional^20^ was utilized. The electron–ion interaction was described using the projector-augmented wave method.^21^ For geometry optimization and electronic structure calculations, we applied an energy cutoff of 500 eV and utilized a Monkhorst–Pack^22^ 3 × 3 × 3 *k*-mesh grid. The lattice constants and atomic positions underwent full relaxation until atomic forces reached magnitudes smaller than 0.01 eV Å^−1^. The convergence criterion for electron relaxation was set at 10^−6^ eV. This methodology ensures accurate and reliable results in accordance with the specified requirements of the experimental study.

## 3. Results and discussion

### 3.1 Characterization of Nb_4_P_2_S_21_ crystal

**Fig. 1:**
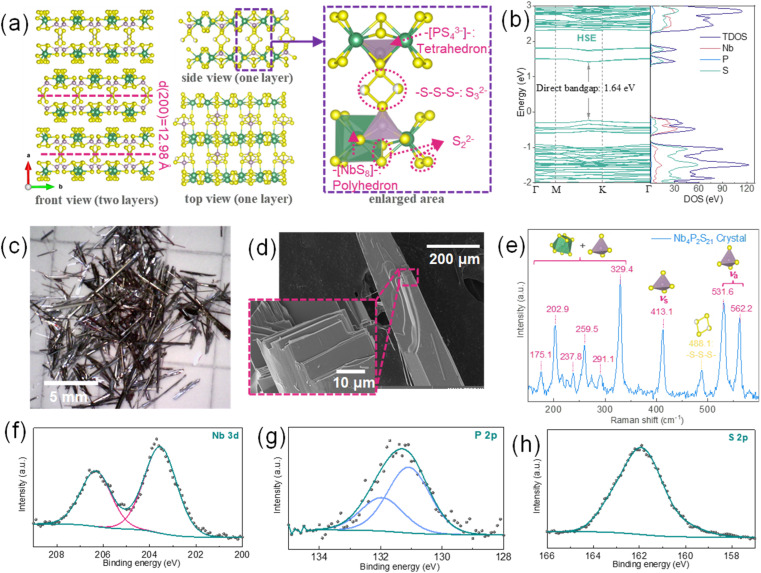
(a) Schematic representation of the crystal structure of Nb_2_P_4_S_21_ showing the front of two layers (left and middle) and side views and the top view of one layer (right), with *d* = 12.84 Å, indicating the interlayer distance. The enlarged area highlights the coordination environment with NbS_8_–NbS_8_ polyhedrons and PS_4_ tetrahedra. (b) Band structure and DOS diagram for Nb_2_P_4_S_21_ calculated using the HSE method. (c) Photograph of prepared Nb_2_P_4_S_21_ crystal. (d) SEM images of an Nb_2_P_4_S_21_ crystal, with the inset showing a higher magnification image revealing the crystal's morphology. (e) Raman spectrum of Nb_2_P_4_S_21_ crystal, displaying characteristic peaks corresponding to various vibrational modes. (f), (g) and (h) X-ray photoelectron spectroscopy (XPS) of Nb 3d, P 2p, and S 2p regions, respectively, with peak fitting indicating the presence of different oxidation states.

As shown in Fig. 1a, Nb_4_P_2_S_21_ crystallizes in the monoclinic C2 space group, having a van der Waals two-dimensional layered crystal structure. The structure is van der Waals two-dimensional. Each Nb atom is bonded to eight S atoms to form NbS_8_ polyhedron unit that shares faces with two other NbS_8_ polyhedra forming a 1D chain structure. Two NbS_8_ units sharing the face with three sulfurs are also connected by PS_4_ unit. Chains are linked together *via* trisulfide (
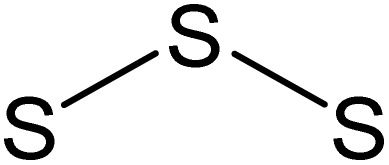
, S_3_) bridges in P_2_S_9_ units creating a single layer of a quasi-1D material. Individual Nb–S and P–S bonds are not equal and their length ranging 2.49–2.71 Å and 2.02–2.17 Å, respectively. The electronic properties of Nb_4_P_2_S_21_ have been investigated using two different exchange–correlation functionals: the HSE and PBE, as shown in Fig. 1b and i, respectively. The HSE functional calculations reveal a direct bandgap of 1.64 eV for Nb_4_P_2_S_21_, indicating semiconductor behavior. The DOS shows sharp peaks near the Fermi level, primarily from Nb 4d and S 3p orbitals, while phosphorus, though structurally significant in PS_4_ tetrahedra, has minimal influence on the bandgap. In contrast, PBE functional calculations show a narrower direct bandgap of 0.81 eV. Both methods indicate direct bandgap characteristics, with similar elemental contributions in the DOS. HSE is considered more accurate for bandgap prediction due to its inclusion of exact exchange, reducing self-interaction errors seen in PBE.^23^

In Fig. 1c, the bulk crystals with a quasi-1D needle morphology are shown, which gives an idea about the highly crystallized Nb_4_P_2_S_21_ single crystal. The SEM image in Fig. 1d reveals the microstructure of the material, and the high magnification inset shows the crystal quality and layered structure, which are swift ion intercalation processes and guest intercalations. Fig. S3† depicts the EDS analysis results of an Nb_4_P_2_S_21_ crystal. The elemental maps for Nb, P, and S show a homogeneous distribution across the crystal. The EDS spectrum quantitatively supports the material composition, revealing a stoichiometry nearly matching the anticipated ratio of Nb : P : S at 4 : 2 : 21. The Raman spectrum of the Nb_4_P_2_S_21_ single crystal is presented in Fig. 1e. The split bands at 531.1 cm^−1^ and 562.2 cm^−1^ are from asymmetric stretching of PS_4_ unit, while symmetric stretching vibration is centered at 413.1 cm^−1^ and swinging vibration is at 329.4 cm^−1^. The signals in the 150–300 cm^−1^ region can be attributed to the symmetric and asymmetric bending modes of PS_4_ tetrahedral overlaps with NbS_8_ polyhedron.^24^ The observation of a signal centred at 488.1 cm^−1^ is indicative of S_3_ bridge bonding within a unit layer.^25^Fig. 1f–h further provides XPS spectra that display the binding states of Nb 3d, P 2p, and S 2p, respectively. Based on the structural analysis of the material described earlier, Nb and P should exhibit single bonding states corresponding to NbS_8_ polyhedral and PS_4_ tetrahedral units, respectively. Nb3d spectrum exhibits a doublet at 203.7 and 206.5 eV that indicates a Nb^4+^ oxidation state.^26^ P2*p* spectrum shows a doublet at 131.2 and 132.0 eV which is typical for PS_4_ tetrahedra. On the other hand, sulfur exhibits a very complex chemical environment, with at least four different binding states expected: Nb_2_**S**, Nb**S**P, P**S**S, and S**S**S. Additionally, there is a significant variety in the Nb–S and P–S bond lengths that can further broaden the S 2p peak. Such a complex nature causes the broadening of S 2p peak which makes it challenging to fit the spectrum correctly since individual states can have very small differences in binding energies (tenths of eV); therefore, the fitting of S 2p was not done. However, the peak is within 159–164 eV which is below the elemental sulfur, thus corresponding to sulfides.

### 3.2 Characterization of exfoliated Nb_4_P_2_S_21_

**Fig. 2:**
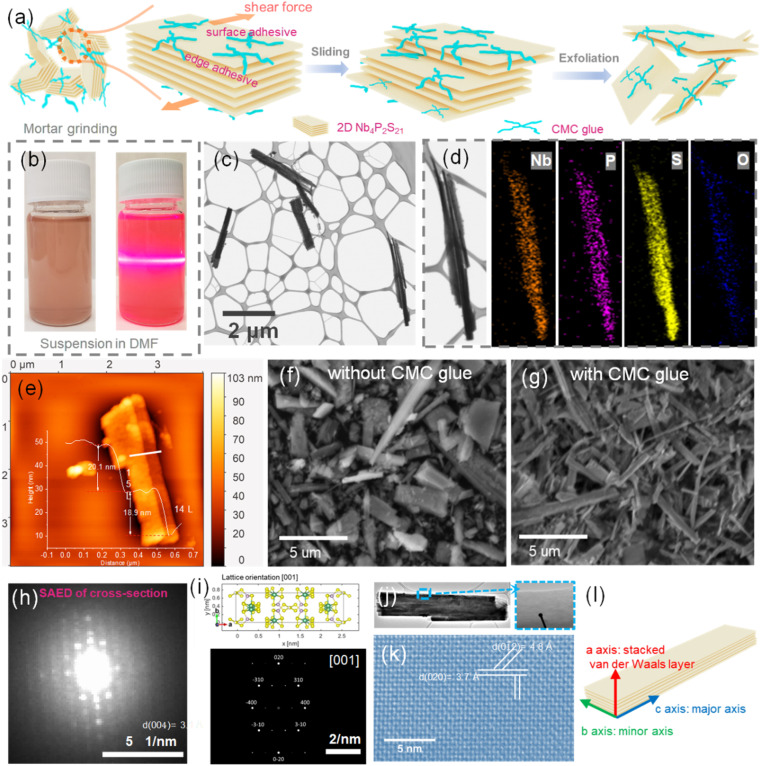
Multifaceted characterization of exfoliated 2D Nb_4_P_2_S_21_ nanoplates. (a) Schematic illustration of the glue-assisted grinding exfoliation process using CMC glue. (b) Photographs showing the dispersion of exfoliated nanoplates in DMF, exhibiting characteristic colors and the Tyndall effect under laser illumination. (c) STEM image of the exfoliated nanoplates. (d) Elemental mapping of selected nanoplates. (e) AFM measurement of larger nanoplates showing varying thickness. (f) SEM images of as-prepared Nb_4_P_2_S_21_ grinding without CMC glue. (g) SEM images of Nb_4_P_2_S_21_ nanoplates with CMC glue-assisted exfoliation. (h) and (i) Low kV electron diffraction analysis of the cross-section of Nb_4_P_2_S_21_. (j) and (k) High-resolution TEM images of exfoliated nanoplates. (l) Schematic representation of the crystal growth structure.

Fig. 2 demonstrates a comprehensive analytical approach to characterizing the structural and optical properties of Nb_4_P_2_S_21_ nanoplates. Fig. 2a provides a mechanistic insight into the exfoliation of bulk Nb_4_P_2_S_21_ using carboxymethyl cellulose (CMC) glue. The CMC, a cellulose derivative with carboxymethyl groups, has a high affinity for the thiophosphate surface due to several functional aspects: (a) surface interaction: the carboxyl groups (–COOH) in CMC can interact with the surface sulfur atoms of the Nb_4_P_2_S_21_ through non-covalent interactions, such as hydrogen bonding. Additionally, the sodium ions present in the CMC structure can engage in ionic interactions with the negatively charged sulfur sites on the material's surface;^27^ (b) Shear forces: during the grinding process, shear forces are generated, which are critical in facilitating the detachment of layers from the bulk material. The CMC glue acts as a medium that transmits these forces effectively to the material. The adhesive nature of CMC means it can “grip” the outermost layers of the Nb_4_P_2_S_21_, allowing the mechanical force applied during grinding to be more effective in peeling off individual layers; (c) layer stabilization: once individual layers are separated, CMC serves to stabilize these newly formed 2D nanoplates by preventing reaggregation. The bulk structure of CMC and its polymeric nature hinder the movement of the exfoliated layers, maintaining them in a dispersed state within the medium. The combined chemical interactions and mechanical forces result in a synergistic effect that enhances exfoliation efficiency. This process leads to the production of thin-layered 2D materials, which are then suspended in the medium, and ready for subsequent applications or further characterizations after cleaning.

Fig. 2b presents exfoliated Nb_4_P_2_S_21_ nanoplates in DMF, showing the dark orange-yellow color of the suspension. The right side under laser illumination exhibits the Tyndall effect, confirming a stable colloidal dispersion of the exfoliated material. Fig. 2c presents a STEM image of exfoliated Nb_4_P_2_S_21_, showing the thin, evenly distributed nanoplates, indicative of a successful exfoliation to a few-layer thickness. The corresponding elemental maps for Nb, P, S, and O in Fig. 2d across a nanoplate, confirm uniform composition and suggest slight surface oxidation, which is typical for such materials when exposed to air. After exfoliation, its EDS spectrum in Fig. S4† exhibits a close stoichiometric ratio of all elements to Nb_4_P_2_S_21_. In addition, no peak for Na Ka at 1.041 was observed, indicating that CMC has been completely removed from the material. Simultaneously, in our sample, we can observe specimens with a lateral size of approximately 300 nm, which exhibit a thickness of ∼1.7 nm in the AFM characterizations shown in Fig. S5.† This thickness indicates a single layer of the multi-atomic layered structure consistent with van der Waals crystals, as demonstrated in Fig. 1a. Furthermore, the elemental mapping presented in Fig. S6† confirms that these thin flake materials are Nb_4_P_2_S_21_. Fig. 2f and g further provides comparative SEM images displaying Nb_4_P_2_S_21_ samples without and with CMC glue during grinding, respectively. The results show Nb_4_P_2_S_21_ without CMC glue for exfoliation, where the material exhibits a bulk morphology with irregularly shaped particles and a broad size distribution. The particles have a more three-dimensional shape, with visible facets and edges, indicating a typical unprocessed bulk material. While the Nb_4_P_2_S_21_ exfoliated with CMC glue, shows the morphology of thinner, more uniform nanoplates, displaying a needle-like or flake-like structure. These structures are characteristic of the delamination effect induced by the exfoliation process, where the CMC glue plays a crucial role. The UV-vis absorption spectra (Fig. S7a†) show two absorption edges for both bulk and exfoliated Nb_4_P_2_S_21_. In the exfoliated sample, edges at 599 nm and 638 nm suggest two electronic transitions: the 599 nm edge likely corresponds to the primary bandgap, while the 638 nm edge may involve excitonic or defect-related transitions. The bulk sample shows edges at 556 nm and 641 nm. Tauc plots estimate an optical bandgap of 2.03 eV for both samples, aligning with HSE-calculated results (Fig. S1b†). Photoluminescence (PL) spectra (Fig. S7b†) reveal a stronger PL peak at 755 nm for exfoliated Nb_4_P_2_S_21_ due to quantum confinement and defect states, while bulk shows a weaker peak at 767 nm, likely due to non-radiative centers.^28^ Raman spectra (Fig. S7c†) indicate shifts in interlayer interactions or lattice strain in exfoliated nanoplates.

While the SEM images of bulk Nb_4_P_2_S_21_ reveal the facets and edges, indicating the layered stacking direction (*i.e.*, the *a*-axis), a more direct confirmation is needed through cross-sectional HRTEM or electron diffraction analysis. We initially attempted to analyze lamellae cut along the major axis of the needle-like Nb_4_P_2_S_21_ for TEM analysis; however, the lamellae in this orientation proved unstable under a high-voltage electron beam, making it challenging to obtain information related to the crystal structure. Consequently, we opted to cut a chunk along the minor axis, which was then lifted out and transferred onto a FIB grid. The sample was further thinned on a compustage within a TF Helios 5 FX dual-beam microscope equipped with a T-Pix detector for electron diffraction collection, as demonstrated in Fig. S8.† The results of the cross-section, shown in Fig. 2h, align well with the simulated material with lattice orientation [001]. The slight rotational offset of the diffraction pattern can be attributed to the manual insertion of the FIB grid into the compustage. This indicates that the needle-like Nb_4_P_2_S_21_ is stacked along the etching direction with van der Waals forces connecting the *a*-axis. In Fig. 2j, k and S9,† HRTEM and SAED analyses were performed on the edges of the exfoliated nanoplatelets, revealing the (020) crystal planes parallel to the long axis and the (004) planes parallel to the short axis. These findings confirm that both the rod-like bulk and exfoliated flake materials are stacked along the *a*-axis direction, with the short *b*-axis and long *c*-axis forming the needle-like Nb_4_P_2_S_21_, as schematically represented in Fig. 2l. Fig. S10a and b† compare the refined XRD spectra of both the bulk powder and the exfoliated material, with both spectra fitting well with the *C*2/*c* space group of Nb_4_P_2_S_21_. However, a slight increase in the lattice parameters of the exfoliated material suggests a reduction in stress and a consequent expansion of the lattice post-exfoliation. When comparing the *I*(400)/*I*(310) crystal plane intensity ratio, the bulk powder exhibits a value of 9.6, larger than the 5.6 observed in the exfoliated nanoplates. In Fig. S10c–i,† we present the positions of the (400) and (310) crystal planes. The reduction in the intensity ratio might be attributed to a relative decrease in material thickness, leading to a reduced number of effective (400) planes along the *a*-axis, as depicted in Fig. S10c-ii and iii.†

### 3.3 Electrochemical reaction analysis of exfoliated Nb_4_P_2_S_21_ nanoplates

In materials with high sulfur content, some electrochemical properties resemble those of elemental sulfur, demonstrating high charge–discharge voltage plateaus and showing promise as alternative cathode materials for sulfur. Moreover, in high voltage ranges under partial discharge conditions, these materials generate a variety of complex products beyond Li_2_S. When the material is fully lithiated, the final product should be similar to that reported in other studies, comprising the corresponding metal elements Nb, Li_2_S, and Li_3_P as the final lithiation products.^8*d*^ The total capacity, based on the complete chemical reaction (Nb_4_P_2_S_21_ + 48 Li → 4Nb + 2Li_3_P + 21Li_2_S), is approximately 1162.6 mA h g^−1^.

**Fig. 3:**
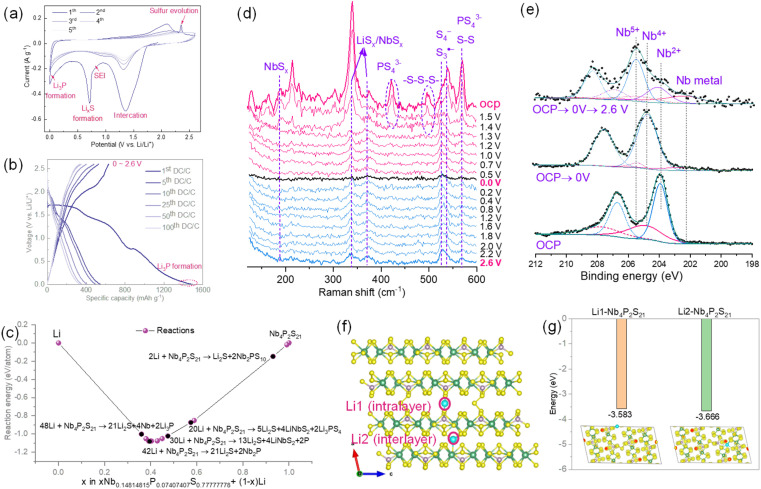
(a) Cyclic voltammetry and (b) discharge–charge over 100 cycles under carbonate-based electrolyte of Nb_4_P_2_S_21_ at potential windows of 0–2.6 V. (c) Generated reactants between Li and Nb_4_P_2_S_21_ with GGA/GGA+*U* (mixed) from the Materials Project. (d) *In situ* Raman at initial cycling under 0–2.6 V. (e) XPS of Nb 3d comparison at the different cut-off voltage. (f) Structure illustration of a single lithium intercalation at intraplane (Li1) and interlayer (Li2) and (g) the corresponding binding energy comparison with inserts of electron density map.

For this material, it is possible to form metal phosphides, metal sulfides, or other types of metal phosphosulfides in different partial discharge ranges. The presence of these *in situ* formed substances may facilitate the conversion of lithium sulfides, achieving excellent electrochemical performance, akin to the role of catalysts in lithium–sulfur battery systems. Therefore, we first assessed the capacity contribution potential of this material across different charge–discharge ranges. As shown in Fig. 3a, the 5 cycles of CV recorded at different voltage ranges begin to exhibit two partially overlapping reduction peaks when the initial reduction voltage is between 1.0–1.7 V. Due to the high intensity and breadth of these peaks and considering the discharge curve from the complete discharge range of 0–2.6 V (Fig. 3b), the discharge capacity within this voltage range approaches half of the total discharge capacity. According to the Interface reactions tools with GGA/GGA+*U* (mixed) from the Materials Project, generating different proportions of reactants (Fig. 3c), we attribute this peak to the combination of lithiation and conversion reactions of Nb_4_P_2_S_21_:Nb_4_P_2_S_21_ + 4 Li → 2LiNb_2_PS_10_ + Li_2_S, (2.0 V → 1.6 V)(16 + 4*x*)Li + Nb_4_P_2_S_21_ → 5Li_2_S + 4Li_*x*_NbS_2_ + 2Li_3_PS_4_Or,(26 + 4*x*)Li + Nb_4_P_2_S_21_ → 13 Li_2_S + 4 Li_*x*_NbS_2_ + 2P, (0 ≤ *x* ≤ 1, 2.0 V → 1.0 V)In the above reaction, LiNb_2_PS_10_ and Li_*x*_NbS_2_ represent the lithiated products of the 2D layered structures of Nb_2_PS_10_ and NbS_2_, as shown in Fig. S11.† The conversion process involving P has been extensively reported.^8*b*,*d*,29^ Additionally, it is noteworthy that prior computational and experimental results indicate that the initial lithium intercalation voltage of NbS_2_ is above 1.5 V, forming LiNbS_2_. Further lithium insertion into LiNbS_2_ converts it to Nb and LiS_2_ at a voltage below 0.5 V.^30^ The theoretical capacity based on the above-mentioned reactions can be calculated as 823 mA h g^−1^ according to Faraday's law (*Q* = (nF)/Mw). When the voltage is further reduced to 0.8 V, the reduction peak can be attributed to the formation of the SEI and the continued lithiation and conversion of Li_*x*_NbS_2_. At 0 V, Li_*x*_NbS_2_ should fully convert to Nb and Li_2_S, with P fully converting to Li_3_P chemically:Li_*x*_NbS_2_ + Li → Nb + Li_2_SP + Li → Li_3_P, (1.0 V → 0 V)In subsequent cycles, a pair of reversible oxidation–reduction peaks at 1.3 V (red.)/2.1 V (ox.) dominates the reaction process, applicable to 0–2.6 V, 0.5–2.6 V, and 1.0–2.6 V ranges. The small peak at 2.35 V during the first oxidation process is attributed to the sulfur evolution process of Li_2_S → 2Li + S, similar to other reported sulfur-rich metal sulfides.^31^ Interestingly, the oxidation–reduction peaks observed in the first cycle remain almost unchanged in subsequent cycles, indicating reversible structural evolution. Although this reversible reaction feature has been reported in similar materials like V_2_PS_10_, its characterization signals after complete lithiation and delithiation in XRD are weak, leaving the reformation of V_2_PS_10_ post-delithiation for further verification.^11*a*^ Consistent with the reported results, when attempting XRD characterization of the electrode material at different stages of the reaction, we observed a strong Li_2_S signal only when discharged to 0 V, with fewer or weaker matching peaks in other charge–discharge states, making it difficult to match the corresponding products, as shown in Fig. S12.† This might be related to the small crystal size of the reaction products or the formation of amorphous metal sulfides. Therefore, we attempted to use *in situ* Raman to characterize changes in the material during the first charge–discharge process.

Within the 0–2.6 V range, although the material initially displays a discharge capacity of up to 1500 mA h g^−1^, the first reversible capacity is only 630 mA h g^−1^, with a rapid capacity fade to about 350 mA h g^−1^ within the first 50 cycles. Additionally, *in situ* EIS spectra within the 0–2.6 V voltage range were recorded, as shown in Fig. S13.† As the material transitions from OCP to a cathodic 1.0 V, the corresponding charge resistance's depressed semicircle gradually decreases, indicating lithiation activation. From 1.0 V to 0.5 V, an additional depressed semicircle in the low-frequency region forms, corresponding to SEI formation. This semicircle continues to enlarge during the anodic scan to 2.6 V and decreases during the cathodic scan to 0 V.

As shown in Fig. 3d, when first discharged to 1.4 V, the vibrational information of the PS_4_ unit and the –S–S–S– linking two Nb_2_PS_10_ quickly disappears, and no observable vibration signal strength appears during the charging process. Instead, the appearance of LiS_*x*_, NbS_*x*_, S–S, S_4_^−^, and S_3_˙^−^ is noted.^32^ The presence of S_3_˙^−^ suggests the equilibrium of Li_2_S_4_ → Li_2_S.^32*b*^ Throughout the entire charge–discharge process, this vibrational peak persists, indicating the continuous conversion process between Li_2_S and polysulfides. Additionally, we recorded the Nb 3d XPS spectra of exfoliated Nb_4_P_2_S_21_ before the electrochemical cycle, after the first discharge to 0 V, and after continuing delithiation charging to 2.6 V, as shown in Fig. 3e. Compared to Nb_4_P_2_S_21_ bulk crystal, exfoliated Nb_4_P_2_S_21_ shows a higher binding energy doublet at 204.9/207.7 eV, possibly due to partial oxidation of the material's surface leading to the formation of Nb^4+^. When discharged to 0 V, mainly two sets of doublets at 204.7/207.5 eV and 202.5/205.3 eV are observed, representing Nb^4+^ and Nb metal, respectively. The former may be due to oxidation during sample transfer or partial non-conversion to metallic NbS_*x*_, while the latter represents the Nb metal formed upon full lithiation. Continuing to charge to 2.6 V, three sets of doublets appear at 205.5/208.3 eV, 204.1/206.9 eV, and 202.4/206.2 eV, representing Nb^5+^, Nb^2+^, and Nb metal, respectively. The presence of Nb^5+^ may be due to the formation of NbS_*x*_ (*x* > 2), similar to XPS results reported in analogous VS_*x*_.^33^ The emergence of Nb^2+^ might be due to the formation of Nb–P–S compounds similar to the initial structure. The existence of Nb metal in this charged state could be due to incomplete reactions preventing Nb from being fully oxidized.

As shown in Fig. 3f, the material is structurally open not only in the van der Waals layer but also in the S_3_ bridge connection layer. These two positions are the most likely sites for the insertion of a single lithium atom into the material. As expected from the analysis, the DFT calculations confirmed that the binding energies of Li1 (S_3_ bridge layer) and Li2 (van der Waals layer) are very close as depicted in Fig. 3g, with values of −3.583 eV and −3.666 eV, respectively. This indicates that lithium atoms can be embedded simultaneously at both sites during the initial stage of lithium insertion.

### 3.3 Performance evaluation as sulfur-equivalent cathode

**Fig. 4:**
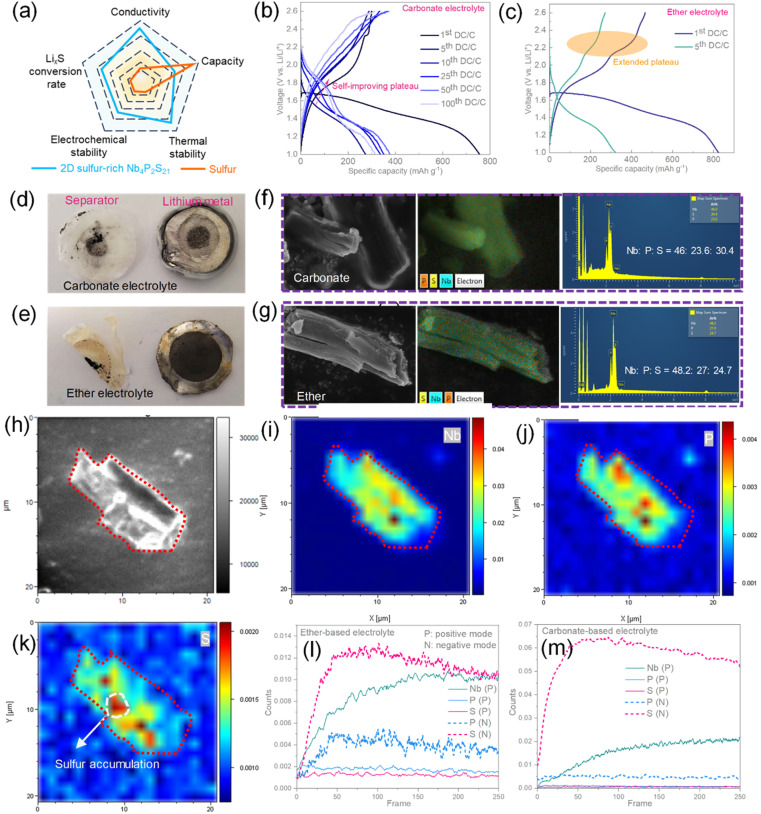
(a) Comparison of sulfur-rich 2D semiconductors and pure sulfur material properties in lithium–sulfur battery system. Charge–discharge curves of Nb_4_P_2_S_21_ under (b) carbonate-based electrolyte and (c) ether-based electrolyte at potential windows of 1.0–2.6 V. (d)–(e) Photographs of separator and lithium metal after 100 cycling under carbonate and ether electrolyte, respectively, and (f)–(g) corresponding SEM-EDS analysis of electrode materials. (h) SEM image of the selected area for post-cycling sample Nb_4_P_2_S_21_ in the ether-based electrolyte. (i–k) SIMS total intensity with the etching depth and (l) depth profiles of elements Nb, P, and S at positive and negative polarity variation under ether-based electrolyte reacted materials. (m) Polarity variation of Nb, P, and S of materials reacted under carbonated-based electrolyte.

Furthermore, we evaluated the long-term charge–discharge capabilities of such materials across different ranges, focusing not only on their potential as high-capacity anodes but more crucially, on assessing whether these sulfur-rich transition metal thiophosphates have the potential, similar to sulfur-rich transition metal sulfides, to serve as promising sulfur-equivalent cathodes. As shown in Fig. 4a, we compared the potential advantages of sulfur-rich materials over pure sulfur. While these materials may have a lower theoretical capacity than pure S, they offer several key benefits: (a) their semiconductor properties provide higher electronic conductivity compared to the insulating nature of pure S; (b) under partial lithiation, intermediate compounds such as transition metal sulfides or phosphides can catalyze the conversion of Li_2_S; (c) the presence of these intermediates can adsorb polysulfides, thereby suppressing the shuttle effect; (d) they also exhibit higher thermal stability.

The long-term charge–discharge curves and recorded cycling data for various ranges under carbonate-based electrolytes are depicted in Fig. 4b and S14.† The test results show that in the 1.0–2.6 V range, the material exhibits a first discharge capacity of 756 mA h g^−1^, with a first reversible capacity of only 300 mA h g^−1^. Interestingly, as cycling progresses, this range also sees a capacity increase and the emergence of a new self-improving discharge plateau around 1.8 V, potentially indicating higher energy density as a cathode material. Over 100 cycles, the material exhibits a reversible capacity of 314 mA h g^−1^, performing better than measured under the potential range of 0.5–2.6 and 1.5–2.6 V (as compared in Fig. S14c†). However, further understanding and improvement are needed regarding the issue of high initial irreversible capacity for these materials to be considered promising sulfur-equivalent cathodes.

In lithium–sulfur battery systems, ether-based electrolytes, rather than carbonate-based ones, are often employed to inhibit the irreversible reaction of carbonates with polysulfides.^34^ Considering the high sulfur content of our material and the presence of polysulfides during the charge–discharge process as indicated by *in situ* Raman, the low reversible capacity observed could be related to the use of carbonate-based electrolytes in the test system. Thus, we conducted electrochemical cycling of the material in a high voltage range using an ether-based electrolyte. As shown in Fig. 4c, under the potential of 1.0–2.6 V, the material demonstrates a higher reversible capacity compared to that in a carbonate-based electrolyte, mainly due to a longer sulfur evolution plateau at 2.2 V, which remains stable through the fifth cycle. However, during extended charge–discharge cycling, the charging curves become extremely unstable, possibly due to the shuttle effect of polysulfides formed during the electrochemical reaction of sulfur-rich materials and the onset of electrode instability.^35^ Further recording of the material's charge–discharge curves at 0.5–2.6 V and 1.5–2.6 V in Fig. S15† reveals that the wider voltage window, the earlier the instability in the charging curves appears. Failure starts from the first cycle in the 0.5–2.6 V window, while in the 1.5–2.6 V window, electrode failure begins after 28 cycles.

Additionally, we disassembled the batteries after 100 cycles in the 1.0–2.6 V window under both carbonate-based and ether-based electrolytes. As shown in Fig. 4d and e, black material is observed on the lithium foil corresponding to the electrode material area in both cases, with a more pronounced and larger black area in the ether electrolyte, and the separator is significantly yellowed. This is because, although ether-based electrolytes can mitigate the side reaction between the electrolyte and polysulfides, they do not address the shuttle effect of polysulfides. This leads to a significant shuttle of active components to the lithium side, causing charging curve oscillations and battery failure. In the carbonate-based electrolyte, there are fewer side reactions on the lithium foil, likely because most formed polysulfides react with the carbonate-based electrolyte on the electrode material side. In Fig. 4f and g, the post-reaction material still appears flaky, but EDS measurements exhibit the element ratios of Nb : P : S is 48 : 27 : 25 reacted under carbonate-based electrolyte and 46 : 24 : 30 under ether-based electrolyte, indicating a significantly lower sulfur content compared to the original material of Nb_4_P_2_S_21_, further verifying the polysulfide shuttle effect similar to the lithium–sulfur system, which major reason for the low reversible capacity and electrode failure. This may also explain why, although we can observe from the SEM that the material maintains its stacked layers after cycling, the crystal structure of the material has undergone significant changes due to the loss of sulfur components.

However, the results of EDS analysis represent a cumulative summation of elemental content. To observe the specific local distribution of elements from the surface to the bulk, TOF-SIMS analysis is required. The TOF-SIMS was utilized to analyze the materials after 100 cycles, examining the etching depth and distribution of elements Nb, P, and S in Nb_4_P_2_S_21_. Fig. 4i–k reveal that the distributions of Nb and P largely overlap; however, significant accumulations of S are evident in certain areas. This segregation of S occurs due to its extraction from the material structure during repeated lithiation processes. The aggregation of S not only leads to poor conductivity in the aggregated areas, rendering the active components underutilized but also exacerbates the shuttle effect, subsequently leading to the failure of the entire battery. Further reducing the material dimensions and adopting strategies similar to those used in lithium–sulfur batteries, such as incorporating adsorptive catalytic media like MXene and C_3_N_4_ mixtures, might effectively unleash the actual energy storage potential of these materials.^36^ The positive polarity profiles presented in Fig. 4l demonstrate a lower concentration of Nb on the surface compared to the bulk, primarily due to the surface being predominantly covered by the SEI layer. Additionally, the signal intensity of P in the positive mode is weak and in the negative mode is much stronger, which is attributed to the original P(5+) in the material being transformed into Li_3_P(3−) during the lithiation process. However, the relative intensity of S from the surface to the bulk is significantly lower than the initial proportion in Nb_4_P_2_S_21_, which further demonstrates the shuttling effect of sulfur components in the ether-based electrolyte. In comparison, as shown in Fig. 4m, the S component in the bulk of the material is a relatively reasonable ratio in the carbonate-based electrolyte. This indicates that the lower sulfur component detected by EDS in Fig. 4f is likely due to the side reactions between the S on the surface and the carbonate electrolyte during the lithiation process, resulting in its loss.

## 4. Conclusions

In this research, we have introduced Nb_4_P_2_S_21_, a novel two-dimensional niobium thiophosphate with a high sulfur content, synthesized *via* a chemical vapor transport and subsequently delaminated into high-quality nanoplates. The material exhibits a layered quasi-1D morphology and a direct bandgap of 1.64 eV, alongside near-infrared photoluminescence at 755 nm. Focusing on LIBs, the electrochemical analysis across different voltage windows reveals that Nb_4_P_2_S_21_ offers promising capabilities as a high-capacity electrode material. Despite its high initial discharge capacity, its performance in carbonate-based electrolytes is compromised by the formation of polysulfides, leading to a shuttle effect that limits reversible capacity and degrades electrode integrity. Transitioning to ether-based electrolytes significantly enhances the reversible capacity of the material by stabilizing sulfur species, although it does not completely overcome the shuttle effect challenges described for lithium–sulfur battery systems. Further post-reaction analyses of the batteries reveal extensive sulfur loss from the active materials and sulfur aggregation within the active materials, and associated irreversible structural changes. These findings suggest that adopting lithium–sulfur battery strategies, such as introducing polar host catalysts, may optimize and enhance the energy storage potential of these materials.

## Data availability

The datasets generated during and/or analyzed during the study are accessible *via* the Zenodo repository: https://zenodo.org/doi/10.5281/zenodo.11501375.

## Conflicts of interest

The authors declare no financial/commercial conflict of interest.

## Supplementary Material

NA-OLF-D4NA01060D-s001
