# Supplementary material for: Glue-assisted exfoliation of two-dimensional sulfur-rich niobium thiophosphate (Nb4P2S21) for sulfur-equivalent electrode study in lithium storage
Source: Nanoscale Adv. 2025 Jan 30;7(7):1860–71. doi: 10.1039/d4na01060d (PMC11804794; doi:10.1039/d4na01060d)
Supplement: NA-007-D4NA01060D-s001 [file NA-007-D4NA01060D-s001.pdf]

Supplementary Information (SI) for Nanoscale Advances.
This journal is © The Royal Society of Chemistry 2025

Supporting Information

Glue-assisted Exfoliation of Two-dimensional Sulfur-rich Niobium Thiophosphate ($Nb_4P_2S_{21}$) for Sulfur-equivalent Electrode Study in Lithium Storage

Bing Wu[1,], Vlastimil Mazánek[1], Min Li[2], Martin Veselý[3], Qiliang Wei[4], Luxa Jan[1], Filipa M. Oliveira[1], Lei Zheng[1], Heng Li[1], Vojtech Kundrat[5], Jakub Zálešák[6], Jakub Regner[1], Rui J. C. Gusmão[1], Junjie He[2], Tomáš Hartman[1], Saeed Ashtiani[1], Yulong Ying[7], Zdenek Sofer[1,*]*

[1] Department of Inorganic Chemistry, University of Chemistry and Technology Prague, Technická 5, 166 28 Prague, Czech Republic
[2] Department of Physical and Macromolecular Chemistry, Faculty of Science, Charles University in Prague, Prague 12843, Czech Republic
[2] Department of Organic Technology, University of Chemistry and Technology Prague, Technická 5, 166 28 Prague, Czech Republic
[4] Institute of Micro/Nano Materials and Devices, Ningbo University of Technology, Ningbo, 315211, P.R. China
[5] Department of Molecular Chemistry and Materials Science, Weizmann Institute of Science, Rehovot 7610001, Israel
[6] Chemistry and Physics of Materials, University of Salzburg, Jakob-Haringer-Strasse 2A, 5020 Salzburg, Austria
[7] School of Materials Science and Engineering, Zhejiang Sci-Tech University, Hangzhou 310018, PR China
* Corresponding Author. E-mail: wui@vscht.cz and zdenek.sofer@vscht.cz

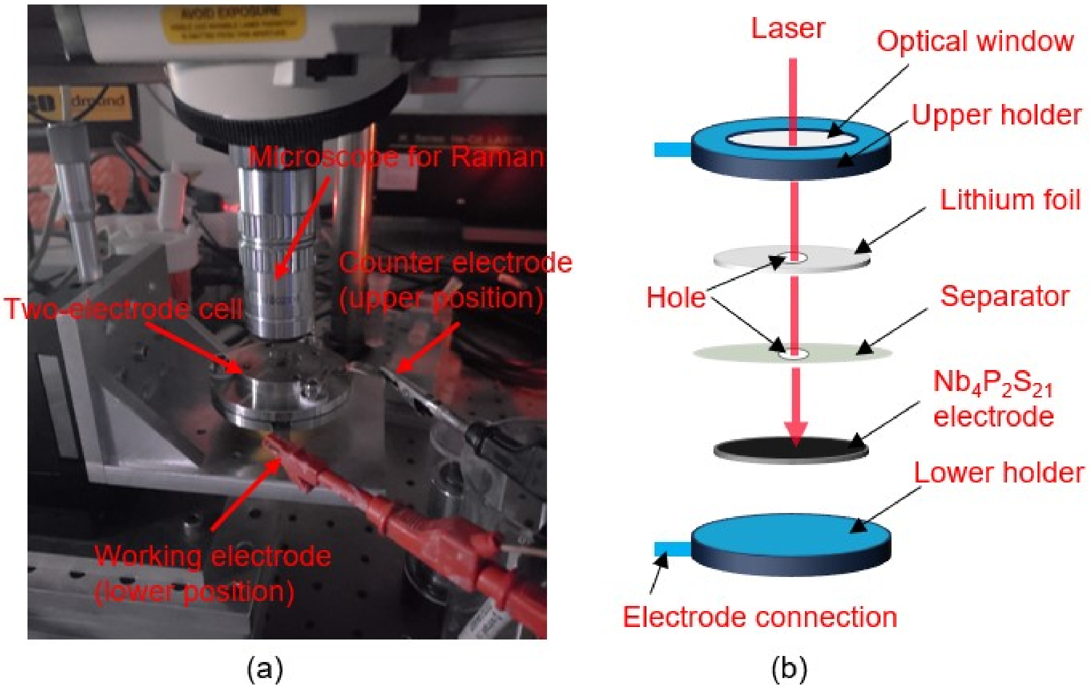

Figure S1. Configuration of the in-situ Raman cell for $Nb_4P_2S_{21}$ electrode analysis. (a) Photograph displaying the connection setup of the cell. (b) Detailed schematic illustration of the individual components within the two-electrode cell.

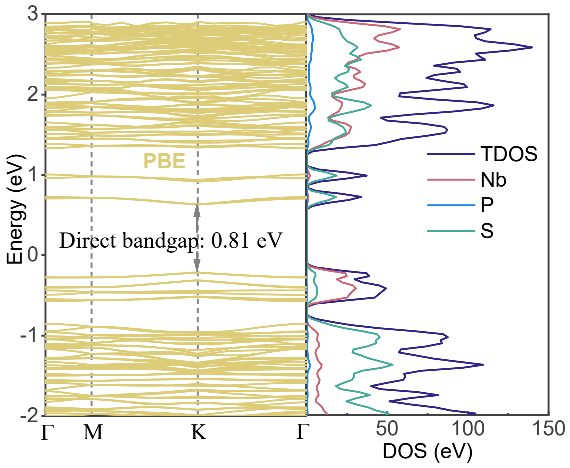

Figure S2 Band structure and density of states (DOS) diagram for $Nb_2P_4S_{21}$ calculated using PBE method.

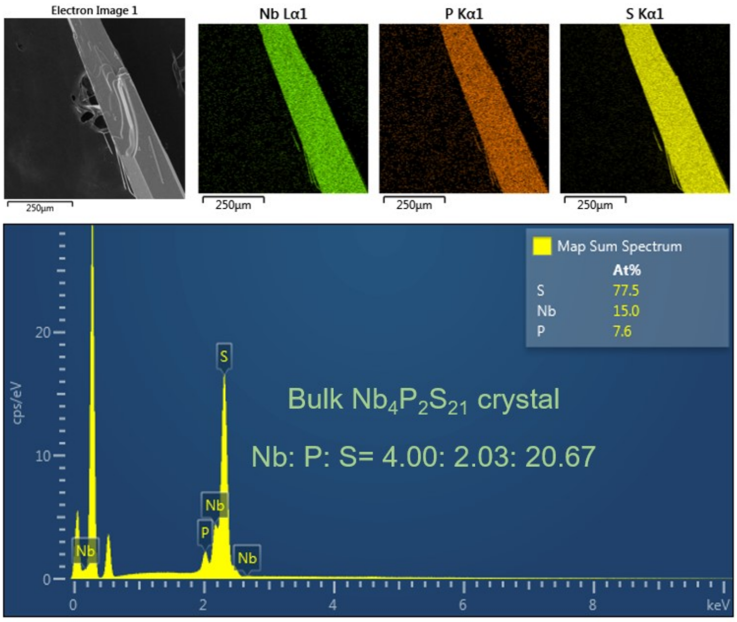

Figure S3 SEM image and EDS analysis of $Nb_4P_2S_{21}$ crystal.

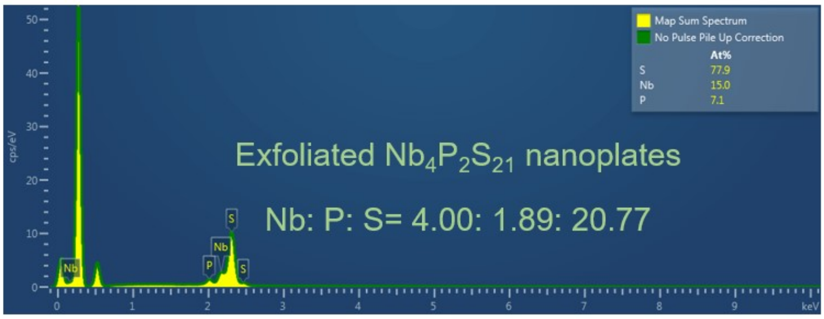

Figure S4 EDS spectrum of exfoliated $Nb_4P_2S_{21}$ nanoplates.

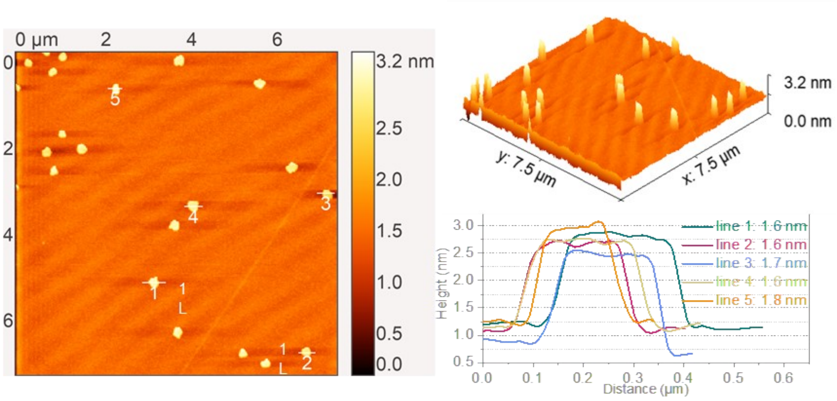

Figure S5 AFM images of smaller, exfoliated nanoplates indicating single-layer thickness.

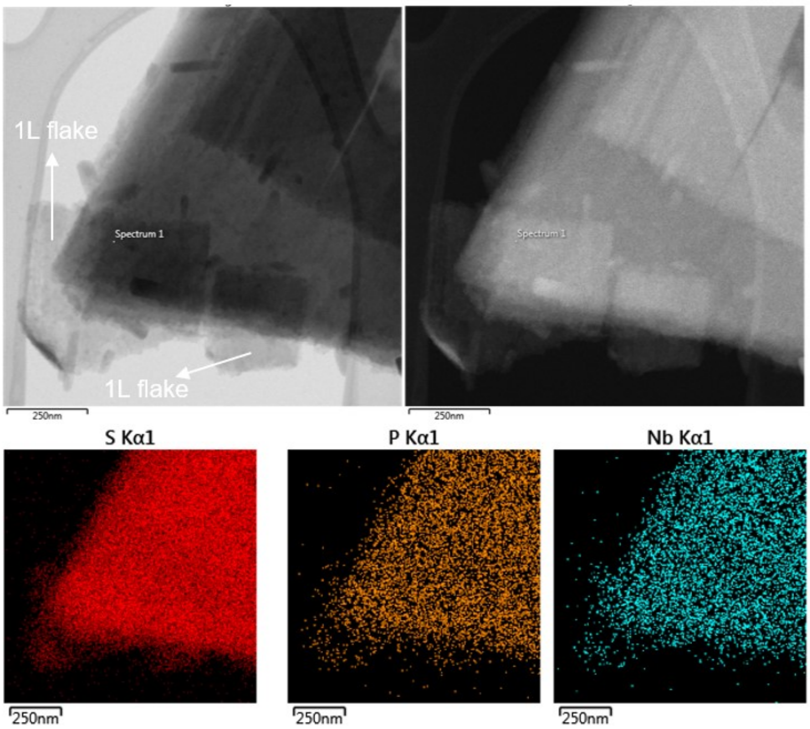

Figure S6 Elemental mapping of exfoliated $Nb_4P_2S_{21}$ nanoplates.

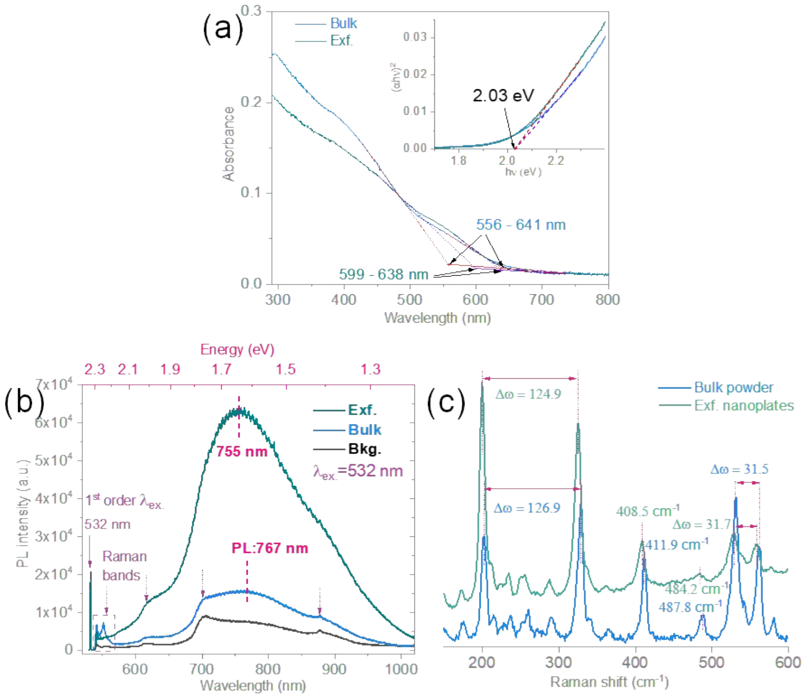

Figure S7 (a) UV-Vis absorption spectra of exfoliated materials and bulk powder with an inset of т plot. (j) Photoluminescence (PL) spectra of exfoliated materials compared to bulk powder. (k) Raman spectra of exfoliated materials versus bulk powder.

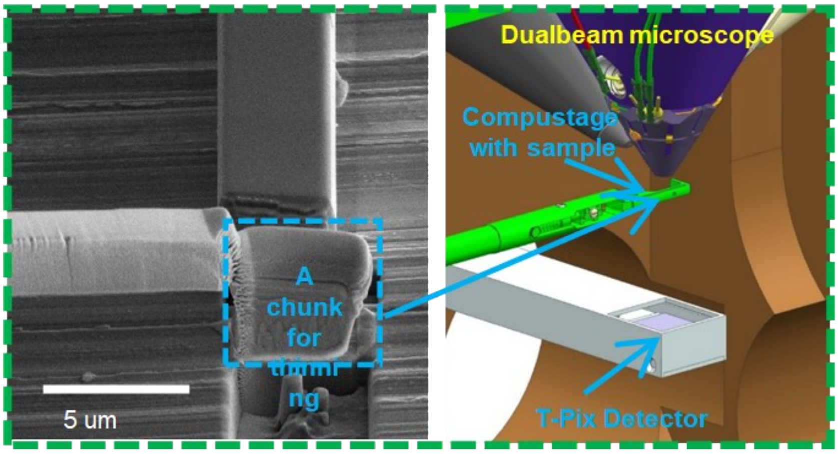

Figure S8 Schematic of performed using a dual-beam microscope equipped with a T-Pix detector for cross-section measurments.

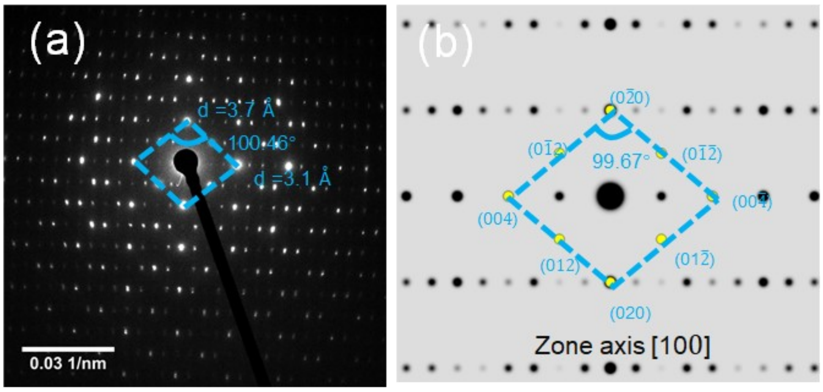

Figure S9 SAED of exfoliated nanoplates with the electron beam oriented perpendicular to the 2D planes.

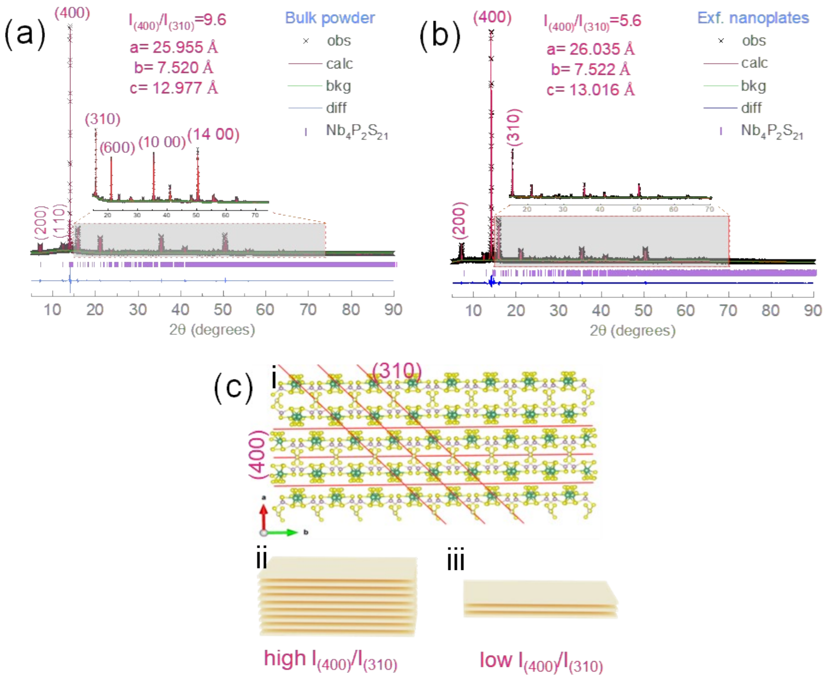

Figure S10 (a-b) XRD refinement results for both bulk powder and exfoliated $Nb_4P_2S_{21}$ nanoplates. (c) Schematic illustrating the relationship between the intensity ratio of the (400)/(310) crystal planes and the thickness of the material.

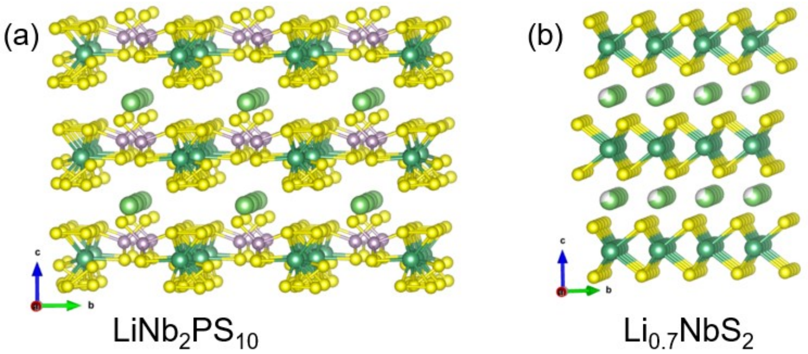

Figure S11 (a) The structure of $LiNb_2PS_{10}$ (The structure originates from substituting K with Li in ICSD#82784 KNb_2PS_{10}). (b) Structure of $Li_{0.7}NbS_2$ (ICSD#281344)

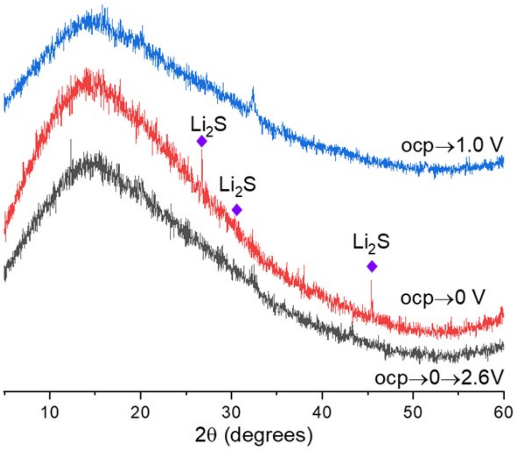

Figure S12 XRD patterns of electrodes discharging from open circuit potential (OCP) to 1.0V, continuously to 0 V and further charging to 2.6 V.

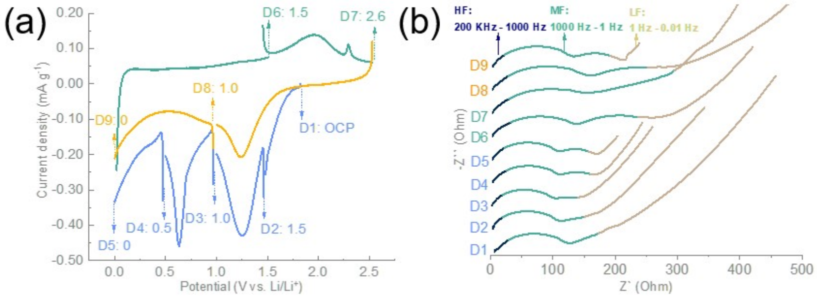

Figure S13 In-situ EIS of $Nb_4P_2S_{21}$ under CV cycling: (a) CV cycling with marked pauses for EIS characterization, (b) EIS spectra recorded at corresponding pauses.

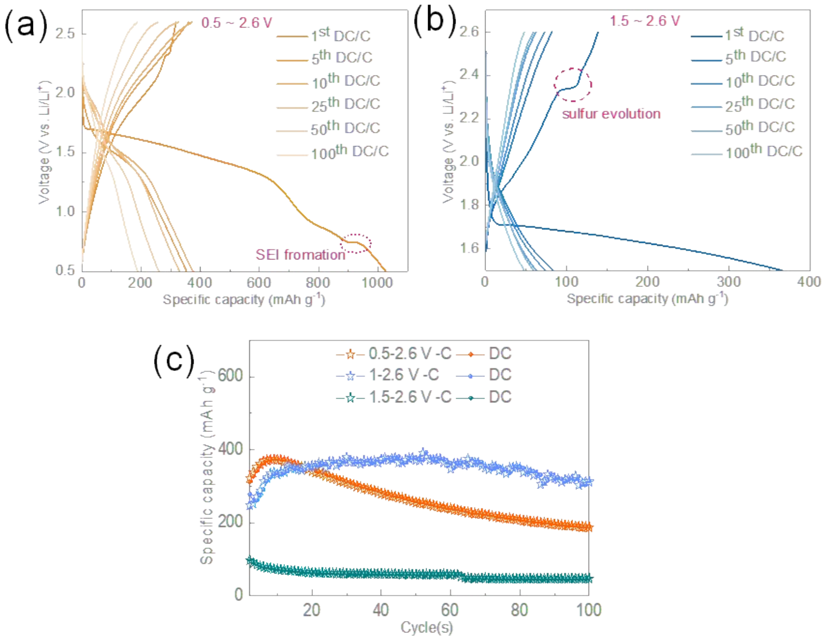

Figure S14 Electrochemical performance of $Nb_4P_2S_{21}$ at potential windows of (a) 0.5-2.6 V, and (b) 1.5-2.6 V, respectively. (c) Corresponding cycling stability from 2nd to 100th cycling.

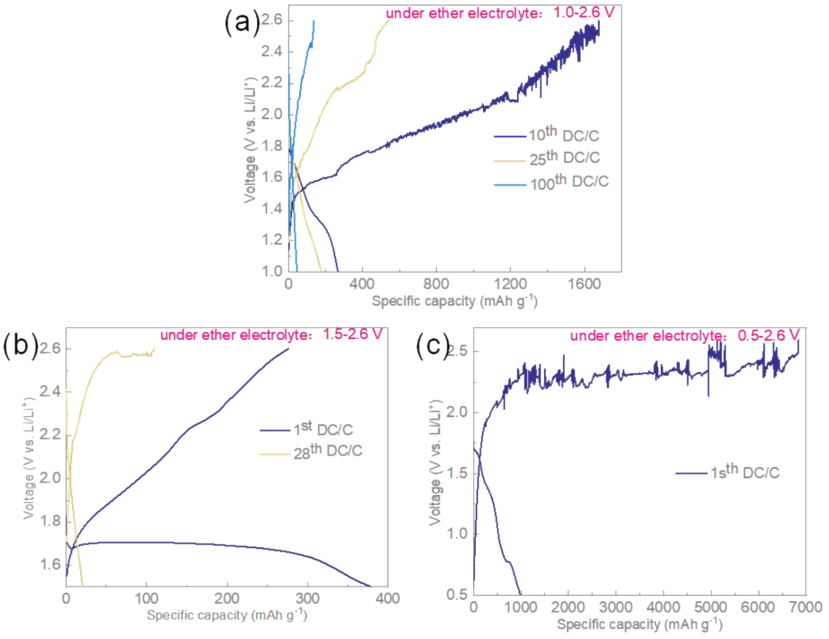

Figure S15 Discharge-charge curves under ether-based electrolyte at (a) 1.0-2.6 V, (b) 1.5-2.6 V, and (c) 0.5-2.6 V.
